# Inhibition of NADPH Oxidase Mediates Protective Effect of Cardiotonic Pills against Rat Heart Ischemia/Reperfusion Injury

**DOI:** 10.1155/2013/728020

**Published:** 2013-06-09

**Authors:** Xiao-Yuan Yang, Na Zhao, Yu-Ying Liu, Bai-He Hu, Kai Sun, Xin Chang, Xiao-Hong Wei, Jing-Yu Fan, Jing-Yan Han

**Affiliations:** ^1^Tasly Microcirculation Research Center, Peking University Health Science Center, Beijing 100191, China; ^2^Department of Integration of Traditional Chinese and Western Medicine, School of Basic Medical Sciences, Peking University, 38 Xueyuan Road, Beijing 100191, China; ^3^Key Laboratory of Microcirculation, State Administration of Traditional Chinese Medicine, Beijing 100191, China; ^4^Key Laboratory of Stasis and Phlegm, State Administration of Traditional Chinese Medicine, Beijing 100191, China

## Abstract

Cardiotonic pill (CP) is a compound Chinese medicine currently used in China for treatment of ischemic angina pectoris. Our previous results indicated that a single dosing of CP pretreatment at 0.8 g/kg attenuates ischemia/reperfusion- (I/R-) induced myocardial injury and cardiac microcirculatory disturbance. The present study aimed to investigate the effect of CP at low dosage in a multiple dosing manner and to uncover the mechanism of antioxidative activity of CP. Male Sprague-Dawley rats were subjected to left anterior descending artery occlusion for 30 min followed by 60 min reperfusion. CP was administrated daily by gavage for six days at 0.1, 0.4, and 0.8 g/kg/day before I/R. Results showed that multiple dosing of CP at three doses significantly reduced I/R-induced myocardial injury, microcirculatory disturbance, and oxidative stress. CP dramatically inhibited I/R-induced nicotinamide adenosine dinucleotide phosphate (NADPH) oxidase subunit gp91^phox^ expression and p67^phox^ and p47^phox^ translocation from cytosol to cell membrane. Translocation of cytosolic subunits to membrane is required for the activation of NADPH oxidase. These data suggested that multiple dosing of CP at doses ranging from 0.1 to 0.8 g/kg/day reduced I/R-induced rat myocardial injury and microcirculatory disturbance, which was mediated by inhibition of NADPH oxidase activation.

## 1. Introduction

Coronary artery disease is a leading cause of death all over the world. Although percutaneous coronary intervention (PCI) is currently used in clinic to restore myocardial perfusion, ischemia/reperfusion- (I/R-) induced injury may lead to cardiac electrical dysfunction and permanent contractile disability. Myocardial injury induced by I/R accounts for the majority of death induced by various forms of cardiovascular diseases [1]. Thus, better understanding the complications of I/R is essential for developing effective treatment to prevent I/R insults [2].

Reactive oxygen species (ROS) plays a crucial role in I/R-induced myocardial damage. Excessive ROS produced by I/R results in increased infarct size and various deleterious events [3]. For instance, ROS directly reacts with membrane lipid, protein, and DNA and eventually causes the damage to cell structure and function [4]. Moreover, massive ROS release leads to activation of transcription factors, such as nuclear factor *κ*B (NF-*κ*B), which in turn result in augmented expression of adhesion molecules and leukocyte infiltration [5]. Inflammation response then concurs to increased extent of tissue injury during I/R. Besides enhancement of inflammation, ROS causes mitochondrial depolarization and prolonged opening of mitochondrial permeability transition pore, triggering cell apoptosis [6]. Given to the central role of ROS in I/R injury, alleviating the oxidative stress is considered to be a potential option in limiting I/R-induced myocardial injury.

Nicotinamide adenosine dinucleotide phosphate (NADPH) oxidase serves as one of the ROS sources in cardiomyocytes, vascular smooth muscle cells, endothelial cells, and fibroblasts [7–10]. Once activated by cytosolic regulatory subunits, NADPH oxidase catalyzes electron transfer from NADPH onto molecular O_2_ to generate O_2_^.−^ [11]. Emerging evidence suggests the involvement of NADPH oxidase in I/R injury. Fukui et al. reported that the expression of NADPH oxidase 2 (NOX2), the catalytic subunit of the phagocyte NADPH oxidase, also known as gp91^phox^, is elevated in rat myocardium after myocardial infarction [12]. Similar results are observed in human myocardium in a subsequent study [13]. Pathogenic roles of NADPH oxidase-derived ROS are also verified in human I/R injury in vivo [14]. NOX2^−/−^ animals with inhibited NADPH oxidase activity exhibit reduced cardiomyocyte apoptosis and improved ventricular function and survival rate after myocardial infarction [15, 16]. Collectively, these results indicate NADPH oxidase as a potential target in treatment of ischemic heart diseases and that pharmacological inhibition of NADPH oxidase might be an efficient strategy to prevent I/R-induced myocardial injury.

Cardiotonic pill (CP), consisting of *Salvia miltiorrhiza* (SM), *Panax notoginseng* (PN), and *Borneol*, is a widely used traditional Chinese medicine (TCM) in China for treating ischemic angina pectoris, which has been scheduled to undergo phase III clinical trials for prevention and treatment of ischemic cardiovascular diseases by the US Food and Drug Administration in 2013. Our previous results showed that a single administration of CP at 0.8 g/kg prevents myocardial damage and apoptosis in rats after 60 min of reperfusion [17], while post-treatment with CP represses I/R-induced myocardial fibrosis in rats [18]. Consistent with the results of CP, accumulating evidence demonstrates the beneficial roles of the components of CP for heart. For instance, SM [19] and its major ingredient, 3,4-dihydroxy-phenyl lactic acid (DLA) [20] and salvianolic acid (SAB) [21], are well established to exhibit antioxidant activity. PN, another ingredient of CP, is reported to attenuate ischemic injury by inhibiting inflammatory response [22]. In spite of this advance, the cardioprotective mechanism of CP is not fully understood at present.

In the present study, we intended to gain further insight into the underlying mechanism of cardioprotection of CP against I/R injury, with particularly focusing on the likely involvement of NADPH oxidase.

## 2. Materials and Methods

### 2.1. Animals

 Male Sprague-Dawley (SD) rats weighing 240–260 g were obtained from the Animal Center of Peking University Health Science Center (Certificate no. SCXK (Jing) 2006-0008). Rats were housed in a humidity of 40% ± 5% and a temperature of 22°C ± 2°C under a 12/12 h light/dark cycle. Rats were free to access water and food, while fasted for 12 h before the surgery. All surgical procedures performed on animals were approved by Peking University Biomedical Ethics Committee Experimental Animal Ethics Branch (LA2010-001), according to the guidelines of the Peking University Health Science Center Animal Research Committee.

### 2.2. Agents

 CP was purchased from Tasly Pharmaceutical Co., Ltd. (Tianjin, China). An analysis by high performance liquid chromatography was carried out for quality control of CP, with one pill containing 9 mg of SM, 1.76 mg of PN, 0.5 mg of *Borneol*, and 13.74 mg of polyethylene glycol. triphenyl tetrazolium chloride (TTC) was obtained from AMRESCO Co., Ltd. (Solon, Ohio, USA) and dissolved in phosphate buffer at a concentration of 0.375%.

### 2.3. Animal Model

SD rats were randomly divided into five groups: Sham, I/R, CP 0.1 + I/R, CP 0.4 + I/R, and CP 0.8 + I/R. Rats were anesthetized by pentobarbital sodium (60 mg/kg, i.p.). Respiration of rats was maintained by a positive-pressure respirator (ALC-V8, Shanghai, China) through an intratracheal cannula. The heart was exposed by a midsternal thoracotomy, and a 5-0 silk ligature was placed around the left anterior descending coronary artery (LAD) [23]. Location of the ligature was 1-2 mm under the boundary of pulmonary conus and left auricle. The LAD was occluded for 30 min to induce myocardial ischemia and then released for reperfusion for 60 min. In Sham group, the rats underwent all the surgical procedures, except for tightening the ligature. Heart tissues were removed at a level 4 mm above the apex after 60 min of reperfusion for determination of the parameters concerned. 

### 2.4. Drug Administration

 CP was dissolved in saline. The animals in the groups pretreated with CP received the drug daily by gavage starting from 6 days before I/R at the dose of 0.1 g/kg/day (CP 0.1 + I/R), 0.4 g/kg/day (CP 0.4 + I/R), or 0.8 g/kg/day (CP 0.8 + I/R), which were 1.2-, 4.9-, and 9.9-fold, respectively, the equivalent effective dose used in clinic. In Sham and I/R group, rats received the same amount of saline in the same way. One and a half hour after the last administration of CP or saline on day 6, animals were anesthetized and subjected to surgical procedure. 

### 2.5. TTC Staining

 At the end of reperfusion, hearts were removed and sliced transversely into five sections (1 mm thick) starting from the ligation site. The slices were incubated in 0.375% TTC at 37°C for 15 min to delineate the infarction area. Slices stained with TTC were analyzed by Image-Pro Plus 5.0 software (Media Cybernetic, Maryland, USA), and infarction was expressed as a percentage of left ventricle area.

### 2.6. Myocardial Blood Flow

 Myocardial blood flow (MBF) was measured by a Laser-Doppler Perfusion Imager (PeriScan PIM3, Perimed, Stockholm, Sweden) equipped with a computer. After left thoracotomy, MBF was recorded at baseline (before ischemia), 30 min after ischemia, and 5, 10, 30, and 60 min of reperfusion, respectively. Acquired MBF images were analyzed by LDPIwin 3.1 software (Perimed, Stockholm, Sweden). MBF results were expressed as percentage of the baseline.

### 2.7. Red Blood Cell Velocity in and Diameter of Coronary Venules

 Coronary venule was observed by an upright microscope (BX51WI, Olympus, Tokyo, Japan) connecting with a high-speed video camera (Photron Fastcam-ultimate APX, Tokyo, Japan) under epi-illumination. The red blood cell (RBC) velocity in the venule was recorded at a rate of 1000 frames/s before ischemia (baseline), 30 min after ischemia, and 30, 60 min of reperfusion, respectively. The stored images were replayed at a rate of 25 frames/s. The venule diameter and RBC velocity were calculated using Image-Pro Plus 5.0 software, as described previously [24].

### 2.8. FITC-Albumin Leakage from Coronary Venules

 At the end of reperfusion, 50 mg/kg of FITC-conjugated albumin (Sigma-Aldrich, St Louis, USA) was injected into the right femoral vein via intravenous catheter. After FITC-albumin injection, venular images were acquired via an upright fluorescence microscope (DM-LFS, Leica, Mannheim, Germany) equipped with a SIT camera (EB-CCD Camera C7190, Hamamatsu, Shizuoka, Japan) under excitation wavelength of 455 nm. The fluorescence intensities of FITC-albumin inside the venules (Iv) and extravenular interstitial area (Ii) were analyzed with Image-Pro Plus 5.0 software. Albumin leakage from coronary venules was expressed by the ratio of Ii/Iv.

### 2.9. Histological and Immunohistochemistry Evaluation of Cardiac Tissues

 Hearts were excised at 60 min of the reperfusion, fixed in 4% paraformaldehyde for 48 h and further prepared for paraffin sectioning. Paraffin sections were stained with hematoxylin and eosin (HE). For immunohistochemistry, the paraffin-embedded sections (5 *μ*m) were rehydrated and treated with 0.01 M sodium citrate for antigen retrieval. After blocked with bovine serum albumin, sections were incubated overnight at 4°C in a humidified box with specific antibodies against Capase-3 (1 : 100, Abcam, Massachusetts, USA), Bcl-2 (1 : 800, Chemicon International, California, USA), Bax (1 : 200, NOVUS, Colorado, USA), NF-*κ*B inhibitor *α* (I*κ*B*α*) (1 : 400, NOVUS, Colorado, USA), and P65 subunit of NF-*κ*B (1 : 700, NOVUS, Colorado, USA). After washed three times by phosphate-buffered saline (PBS), sections were incubated with a Horse Radish Peroxidase (HRP) conjugated secondary antibody and revealed using the diaminobenzidine (DAB) substrate kit. For each section, five fields were selected from the surrounding infarction areas of the left ventricle and captured at a magnification ×200 by a conventional microscope (Digital Sight DS-5M-U1, Nikon, Tokyo, Japan) connected with a digital camera (SZ-40, Olympus, Tokyo, Japan). Mean density was determined by Image-Pro Plus 5.0 software.

### 2.10. Detection of Cardiomyocyte Apoptosis

 At the end of reperfusion, rat hearts were excised and prepared for paraffin sections as described above. Section was stained with terminal deoxynucleotidyl transferase-mediated dUTP nick end labeling (TUNEL) by using a cell death detection kit (Roche, Basel, Switzerland), according to the manufacturer's protocol. Five visual fields were captured from the surrounding infarction areas of the left ventricle by a laser confocal microscope (Axiovert 200M, Carl Zeiss, Jena, Germany). The number of total nuclei and the TUNEL-positive nuclei in each field were counted and analyzed with Image-Pro Plus 5.0 software.

### 2.11. Myocardial Ultrastructure

 Hearts were fixed by infusion with a mixture of 5% paraformaldehyde and 2% glutaraldehyde for 40 min after reperfusion. Then, cardiac tissue was excised from the ischemic region of left ventricle and trimmed into a block 1 mm^3^. The tissue block was then fixed overnight in 3% glutaraldehyde at 4°C and postfixed by 1% osmium tetroxide for 2 h. Tissues were prepared as routing for ultrathin sectioning. Sections were stained with uranyl acetate and lead citrate, then observed using a transmission electron microscope (JEM 1230, JEOL, Tokyo, Japan).

### 2.12. Western Blot

 Myocardial tissues were collected from the ischemic areas of the left ventricle and then homogenized. Whole protein was extracted using a protein extraction kit (Applygen Technologies, Beijing, China) and mixed with 5x electrophoresis sample buffer. Cytosol proteins and membrane proteins were separated by Nucl-Cyto-Mem Preparation Kit (Applygen Technologies, Beijing, China), according to the manufacturer's protocol. After separated by SDS-polyacrylamide gel electrophoresis (SDS-PAGE), proteins were transferred to polyvinylidene difluoride (PVDF) membrane. Nonspecific binding sites were blocked with 5% skimmed milk in Tris-buffered saline Tween (TBS-T). Then, PVDF membranes with target membrane proteins were incubated overnight at 4°C with primary antibodies against gp91^phox^ (1 : 2000, Abcam, Massachusetts, USA), p67^phox^ (1 : 800, Abcam, Massachusetts, USA), p47^phox^ (1 : 200, Santa Cruz Biotechnology, California, USA), p40^phox^ (1 : 200, Santa Cruz Biotechnology, California, USA), and GAPDH (1 : 3000, Cell Signaling Technology, Vermont, USA). The PVDF membranes with target cytosolic proteins were incubated overnight at 4°C with primary antibodies against p67^phox^ (1 : 800, Abcam, Massachusetts, USA), p47^phox^ (1 : 200, Santa Cruz Biotechnology, California, USA), p40^phox^ (1 : 200, Santa Cruz Biotechnology, California, USA), and *β*-tubulin (1 : 3000, Cell Signaling Technology, Vermont, USA). After washing three times by TBS-T, PVDF membranes were then incubated with HRP-conjugated secondary antibody (1 : 5000, Cell Signaling Technology, Vermont, USA) for 1 h at room temperature. Antibody bindings were revealed using Enhanced Chemiluminescence (ECL) Detection Kit (APPLYGEN, Beijing, China). For quantification, band intensity was assessed via Bio-Rad Quantity One software (Bio-Rad, California, USA).

### 2.13. Determination of Malondialdehyde, Superoxide Dismutase, Catalase, and Glutathione Level

 Rats were sacrificed after 60 min of reperfusion; infarcted myocardial tissues were dissected from left ventricle. Immediately after snap frozen in liquid nitrogen, tissues were stored at −80°C till use. Whole proteins were extracted using a protein extraction kit (Applygen, Beijing, China), and protein concentration was determined with BCA protein assay kit (Applygen, Beijing, China), according to manufacturer's instruction. The level of malondialdehyde (MDA), superoxide dismutase (SOD), catalase (CAT), and glutathione (GSH) were determined by using MDA Detection Kit (Nanjing Jiancheng Institute of Biotechnology, Nanjing, China), SOD Assay (R&D, Minnesota, USA), CAT ELISA Kit (R&D, Minnesota, USA), GSH ELISA Kit (R&D, Minnesota, USA), respectively. All tests were performed twice according to manufacturer's instruction. Plates were analyzed on MULTISKAN MK3 enzyme microplate reader (Thermo Fisher Scientific Inc., Illinois, USA).

### 2.14. Expression of CD18 and CD11b on Neutrophils

 Ten milliliters of blood were taken from the abdominal aorta of rats after 60 min of reperfusion. Blood was anticoagulated with 3.8% sodium citrate at a ratio of 9 : 1 (v/v). Fifty microliters of blood were then incubated with FITC-labeled anti-CD18 antibody (5 *μ*g/mL) (BD, Franklin Lakes, NJ, USA) and FITC-labeled anti-CD11b antibody (5 *μ*g/mL) (BD, Franklin Lakes, NJ, USA) for 20 min at room temperature in the dark. The erythrocytes were lysed with hemolysin (BD, New Jersey, USA), according to the manufacturer's instruction. After washing twice by PBS, mean fluorescence intensity was measured using a flow cytometer (FACSCalibur, BD Company, New Jersey, USA). Five thousand neutrophils were sorted and analyzed for each sample as reported previously [17].

### 2.15. Peroxide in Neutrophils

 During reperfusion, a hydrogen peroxide (H_2_O_2_) sensitive fluorescent probe dihydrorhodamine 123 (DHR) dissolved in 3 mL saline was infused into femoral vein via intravenous catheter (2 *μ*mol/kg). At the end of reperfusion, blood was collected from the abdominal aorta of rats and anticoagulated with heparin. Fluorescence intensity of DHR was determined using a flow cytometer (FACS Calibur, BD Company, New Jersey, USA) at excitation light of 510 nm and emission wavelength of 534 nm.

### 2.16. Statistical Analysis

 All data were expressed as mean ± SEM statistical analysis adopted SPSS 11.5 statistical package and was performed using one-way ANOVA followed by Newman-Keuls test or using two-way ANOVA followed by Bonferroni for multiple comparisons. A *P* value less than 0.05 was considered to be statistically significant.

## 3. Results

### 3.1. CP Administration Diminishes I/R-Induced Infarct Size

**Figure 1:**
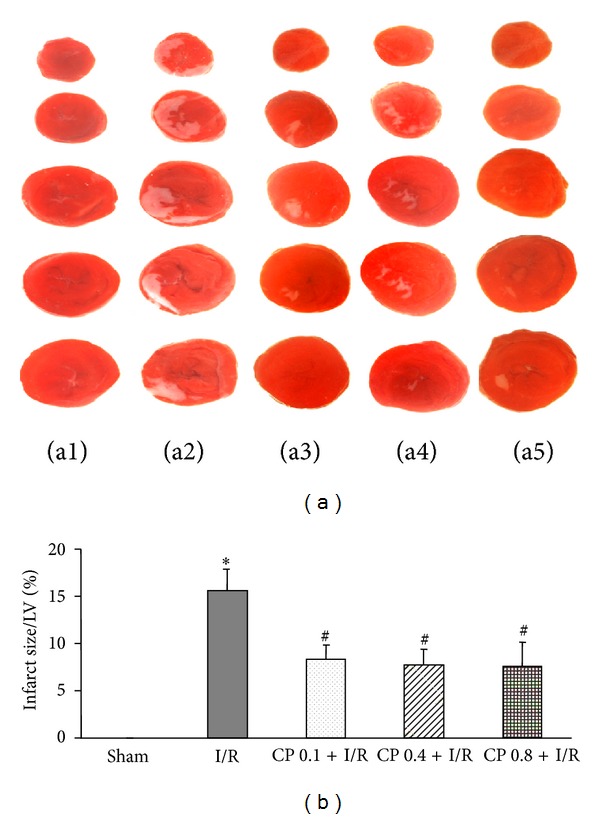
CP pretreatment reduces I/R-induced infarct size. (a) Representative images of myocardial tissue slices stained with TTC in Sham group (a1), I/R group (a2), CP 0.1 + I/R group (a3), CP 0.4 + I/R group (a4), and CP 0.8 + I/R group (a5). White territory represents infarct area. (b) Quantitative results of infarct size in each group. Sham: Sham group; I/R: ischemia/Reperfusion group; CP 0.1 + I/R: CP pretreatment at 0.1 g/kg/day for 6 days plus I/R operation; CP 0.4 + I/R: CP pretreatment at 0.4 g/kg/day for 6 days plus I/R operation; CP 0.8 + I/R: CP pretreatment at 0.8 g/kg/day for 6 days plus I/R operation. The treatment of animals in each group is detailed in Section 2. Results are presented as mean ± SEM (*n* = 6).*P < 0.05 versus Sham group,^#^ *P* < 0.05 versus I/R group.

 Rat hearts were subjected to 30 min of left descending artery occlusion followed by 60 min of reperfusion. Infarct size of various groups was detected to evaluate the cardioprotective role of CP. Representative heart slices stained by TTC to delineate infarct size are shown in Figure 1(a). Apparently, no infarct was observed in myocardial tissue slices from Sham group. However, myocardium sections from I/R group exhibited obvious infarct areas, which were protected by administration of CP at the three doses tested. Quantitative analysis of the infarct size further confirmed that the hearts from CP treated rats showed significantly smaller infarct size compared to those from I/R group, suggesting that CP administration exerted beneficial effects on I/R-challenged myocardium (Figure 1(b)).

### 3.2. CP Administration Inhibits I/R-Induced Myocardial Apoptosis

**Figure 2:**
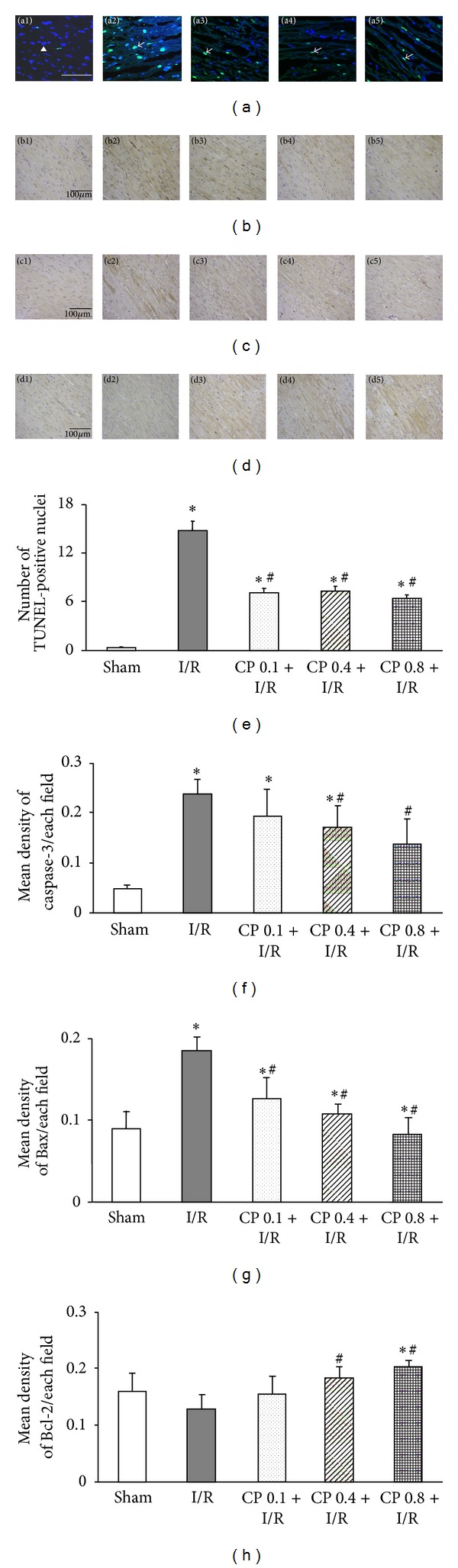
The effects of CP pretreatment on I/R-induced apoptosis and the expression of apoptosis related proteins. (a) Representative images of TUNEL stained myocardial sections in various groups. Apoptotic nuclei are indicated by TUNEL staining (green), and total nuclei are identified by Hoechst staining (blue). Arrowheads indicate normal nuclei. Arrows indicate TUNEL positive cells. Bar = 50 *μ*m. (b) Representative photographs of immunohistochemistry staining for Caspase-3 in different groups. (c) Representative images of myocardial slices stained for Bax in different groups. (d) Representative slices of immunohistochemistry staining for Bcl-2 protein in various groups. Immunohistochemistry positive staining cells are shown in brown color. Bar = 100 *μ*m. (e) to (h) Shown are statistical analysis of TUNEL positive nuclei, Caspase-3 expression, Bax expression, and Bcl-2 expression, respectively, in five different groups. 1, 2, 3, 4, and 5 which denotes Sham group, I/R group, CP 0.1 + I/R group, CP 0.4 + I/R group, and CP 0.8 + I/R group, respectively. Data are presented as mean ± SEM (*n* = 6). *P < 0.05 versus Sham group, ^#^ *P* < 0.05 versus I/R group.

 To investigate the effect of pretreatment with CP on myocardial apoptosis upon I/R, TUNEL staining and immunohistochemistry of apoptosis-related protein were conducted on myocardium from different groups 60 min after reperfusion.  Figure 2(a) illustrates the representative images of left ventricular myocardium stained with TUNEL in surrounding infarct areas from various groups. Only few TUNEL-positive cells were observed in Sham group. In contrast, numerous TUNEL-positive cells were detected in I/R group, which were remarkably reduced in CP pretreated myocardium. Consistent with confocal survey, statistical results also indicated that the hearts from CP pretreated rats showed a significant decrease in the number of TUNEL-positive cells compared to those from I/R group after I/R (Figure 2(e)).

Apoptosis related proteins play a pivotal role in mediating myocardium apoptosis, thus expression of Caspase-3, Bax, and Bcl-2 was assessed by immunohistochemistry in the present study.  Figure 2(b) shows the representative immunohistochemistry images of Caspase-3 in all groups. Only a few cells exhibited Caspase-3 positive staining in Sham group, whereas overexpression of Caspase-3 was detected in I/R group. Corresponding statistical results demonstrated that this increased expression of Caspase-3 was dramatically depressed by pretreatment with CP at 0.4 g/kg/day or 0.8 g/kg/day, but not at 0.1 g/kg/day (Figure 2(f)). Similar to the results of Caspase-3, representative pictures (Figure 2(c)) and quantitative analysis (Figure 2(g)) of Bax showed that administration of CP at all three doses markedly reversed upregulated level of Bax induced by I/R. On the contrary, expression of Bcl-2 was decreased significantly after I/R challenge, which was restored by pretreatment with CP at 0.4 g/kg/day or 0.8 g/kg/day, but not at 0.1 g/kg/day (Figures 2(d) and 2(h)). These results suggested that CP exerted its antiapoptosis role by reducing the expression of proapoptotic protein Caspase-3 and Bax and promoting the expression of anti-apoptotic protein Bcl-2 as compared to rats in I/R group.

### 3.3. CP Administration Restores RBC Velocity in Coronary Venules after I/R

**Figure 3:**
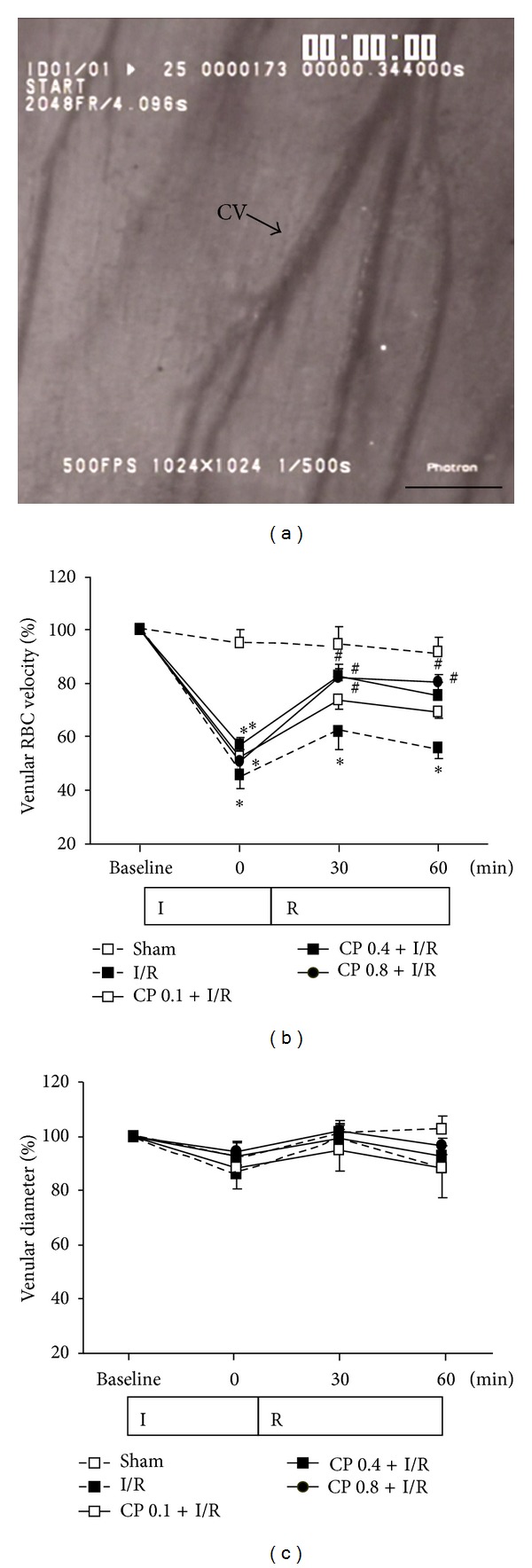
Effect of CP pretreatment on RBC velocity and venular diameter. (a) Representative image of cardiac coronary microcirculation. Bar = 100 *μ*m. (b) Time course of coronary venular RBC velocity in various groups. Results of RBC velocity are expressed as a percentage of baseline. (c) Quantitative results of coronary venular diameter. Results are presented as mean ± SEM (*n* = 6). *P < 0.05 versus Sham group, ^#^ *P* < 0.05 versus I/R group.

 Coronary venules and RBC movement inside the coronary venules of a beating heart were clearly recorded by a high-speed video camera (Figure 3(a)).  Figure 3(b) illustrates the time course of changes in RBC velocity in coronary venules during I/R in various groups. No obvious difference was detected at baseline among groups. However, marked decrease in RBC velocity in I/R group was observed from the end of ischemia till 60 min of reperfusion, compared with Sham group. Pretreatment with CP significantly restored the decline in RBC velocity induced by I/R.

Statistical analysis in Figure 3(c) showed that diameters of coronary venules at baseline were comparable among the five groups, and no significant alteration in the coronary venule diameters of all groups was detected throughout the whole observation period.

### 3.4. CP Administration Prevents Albumin Leakage from Coronary Venules after I/R

**Figure 4:**
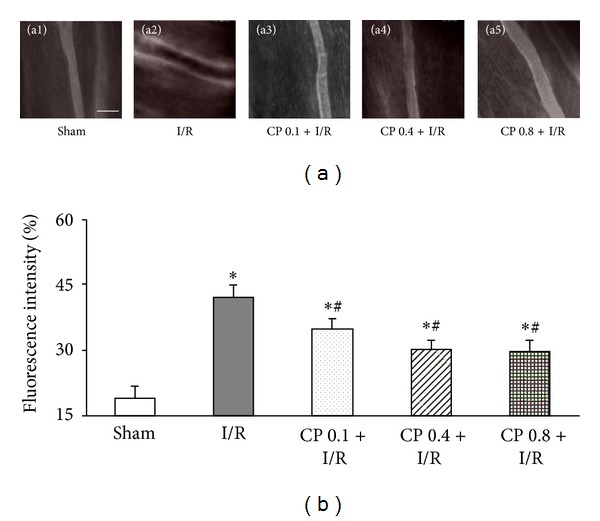
CP pretreatment prevents coronary venular albumin leakage. (a) Representative images of albumin leakage from coronary venules in Sham group (a1), I/R group (a2), CP 0.1 + I/R group (a3), CP 0.4 + I/R group (a4), and CP 0.8 + I/R group (a5). Bar = 100 *µ*m. (b) Statistical results of albumin leakage expressed as ratio of fluorescence intensity in interstice to that in venular lumen. Results are presented as mean ± SEM (*n* = 6). *P < 0.05 versus Sham group, ^#^ *P* < 0.05 versus I/R group.

 Representative fluorescence images of coronary venules in Figure 4(a) showed that no evident FITC-labeled albumin leakage was detected in Sham group, whereas, obvious leakage was visible in the rats from I/R group, which was markedly reversed by CP pretreatment at all three doses. Likewise, quantified results in Figure 4(b) have shown that I/R group displayed significantly up-regulated albumin leakage from coronary venules as compared to Sham group, while pretreatment with CP remarkably prevented albumin leakage from coronary venules induced by I/R.

### 3.5. CP Administration Preserves Myocardial Blood Flow after I/R

**Figure 5:**
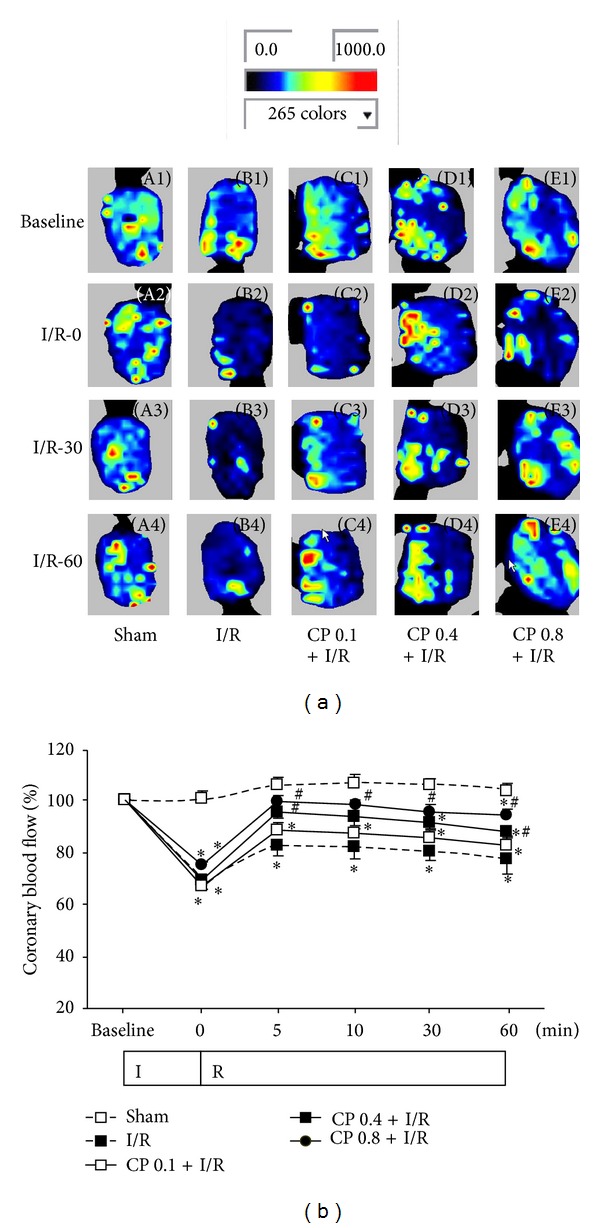
CP pretreatment improves MBF. (a) Representative MBF images acquired by Laser Doppler Perfusion Imager in Sham group (A), I/R group (B), CP 0.1 + I/R group (C), CP 0.4 + I/R group (D), and CP 0.8 + I/R group (E). For each group, images at baseline (1) and 0 min (2), 30 min (3), and 90 min of reperfusion (4) are shown, respectively. Color scale illustrates myocardial blood flow from dark blue (low flow) to red (high flow). (b) Time course of quantitative results of MBF in various groups. MBF is expressed as a percentage of baseline MBF. Data are presented as mean ± SEM (*n* = 6). *P < 0.05 versus Sham group, ^#^ *P* < 0.05 versus I/R group.

 Recovery of RBC velocity in coronary venules and microvascular endothelium barrier suggested that CP pretreatment might improve myocardial coronary perfusion upon I/R. To verify this presumption, MBF was assessed by using a Laser Doppler Perfusion Imager. Acquired representative pictures of MBF at different time points of I/R in all groups are illustrated in Figure 5(a). As shown in Figure 5(b), the time courses of quantitative evaluation of MBF demonstrated that MBF fell dramatically after 30 min of ischemia compared with Sham group; however, this decrease in MBF during I/R was remarkably recovered by pretreatment with CP at 0.4 g/kg/day and 0.8 g/kg/day. Lower dose (0.1 g/kg/day) of CP showed no effect on MBF after I/R.

### 3.6. CP Administration Retains Myocardium Structure after I/R

**Figure 6:**
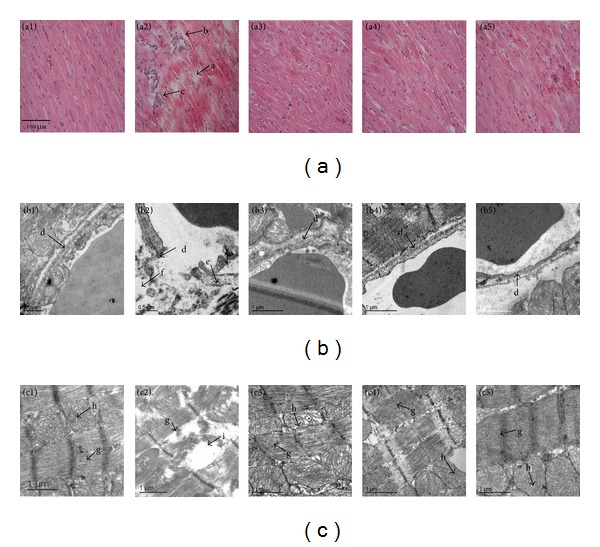
CP pretreatment diminishes I/R-induced alteration in myocardial tissue morphology. (a) Representative images of myocardial sections stained with HE. a: Disrupted myocardial fiber. b: Interstitial edema. c: Inflammatory cell infiltration. Bar = 100 *μ*m. (b) Representative electron micrographs of myocardial capillary from various groups. d: Vascular endothelium. e: Caveolae. f: Interstitial edema. (c) Representative electron micrographs of myocardial fiber in different groups. g: Myofilament. h: Mitochondria. i: Corrupted mitochondria. Results are presented for Sham group (1), I/R group (2), CP 0.1 + I/R group (3), CP 0.4 + I/R group (4), and CP 0.8 + I/R group (5).

 Micrographs of HE stained myocardial sections in various groups are presented in Figure 6(a), revealing that distinct morphological injury, such as myocardial fiber disarrangement and disruption, myocardial tissue edema, and leukocyte infiltration, occurred in infarction areas of myocardial tissues from rats exposed to I/R. Noticeably, pretreatment with CP significantly preserved myocardium structure after I/R (Figure 6(a)).

The representative ultrastructure micrographs of myocardium are displayed in Figure 6.  Figure 6(b) has shown the representative images of capillary from infract region of myocardial tissues. Capillary and its surrounding tissues in Sham group displayed normal ultrastructural feature, whereas I/R challenge evoked capillary endothelium damage, caveolae augment; and perivascular edema. CP pretreatment well preserved capillary ultrastructure of myocardium after I/R. 

Figure 6(c) shows the representative photographs of myocardial cells from infract region of myocardial tissues. In Sham group, myocardial cells retained regularly arranged myofilaments and mitochondria with densely packed cristae. Exposure to I/R led to a dramatic myocardium ultrastructure alterations, such as disrupted myofibrils and disordered mitochondrial cristae. This myocardium injury induced by I/R was markedly alleviated by pretreatment with CP, particularly at dose of 0.8 g/kg/day.

### 3.7. CP Administration Inhibits Oxidative Stress Induced by I/R

**Figure 7:**
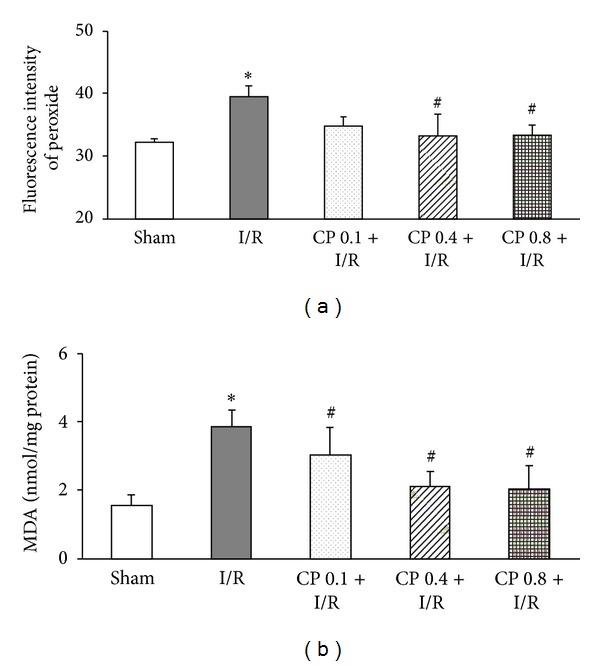
CP pretreatment alleviates I/R-induced oxidative stress. (a) Peroxide levels in peripheral blood are presented as fluorescence intensity of peroxide sensitive probe DHR in different groups. (b) MDA level of myocardial tissue in various groups. Data are presented as mean ± SEM (*n* = 6). *P < 0.05 versus Sham group, ^#^ *P* < 0.05 versus I/R group.

 To investigate oxidative stress, peroxide in blood was detected by using a H_2_O_2_ sensitive fluorescent probe DHR. The levels of peroxide indicated by fluorescence intensity are illustrated in Figure 7(a). I/R provoked a pronounced increase in peroxide production after 60 min of reperfusion, as compared to Sham group. Pretreatment with CP at dose of 0.4 g/kg/day and 0.8 g/kg/day markedly attenuated I/R-induced peroxide enhancement.

Furthermore, myocardium MDA, an indicator of cellular lipid peroxidation, was also measured in different groups. As shown in Figure 7(b), the level of MDA in I/R group was significantly elevated compared with that in Sham group. The increased production of MDA induced by I/R was dramatically blunted by pretreatment with CP at the three doses examined.

### 3.8. CP Administration Inhibits NF-*κ*B Activation and Neutrophil Adhesion Induced by I/R

**Figure 8:**
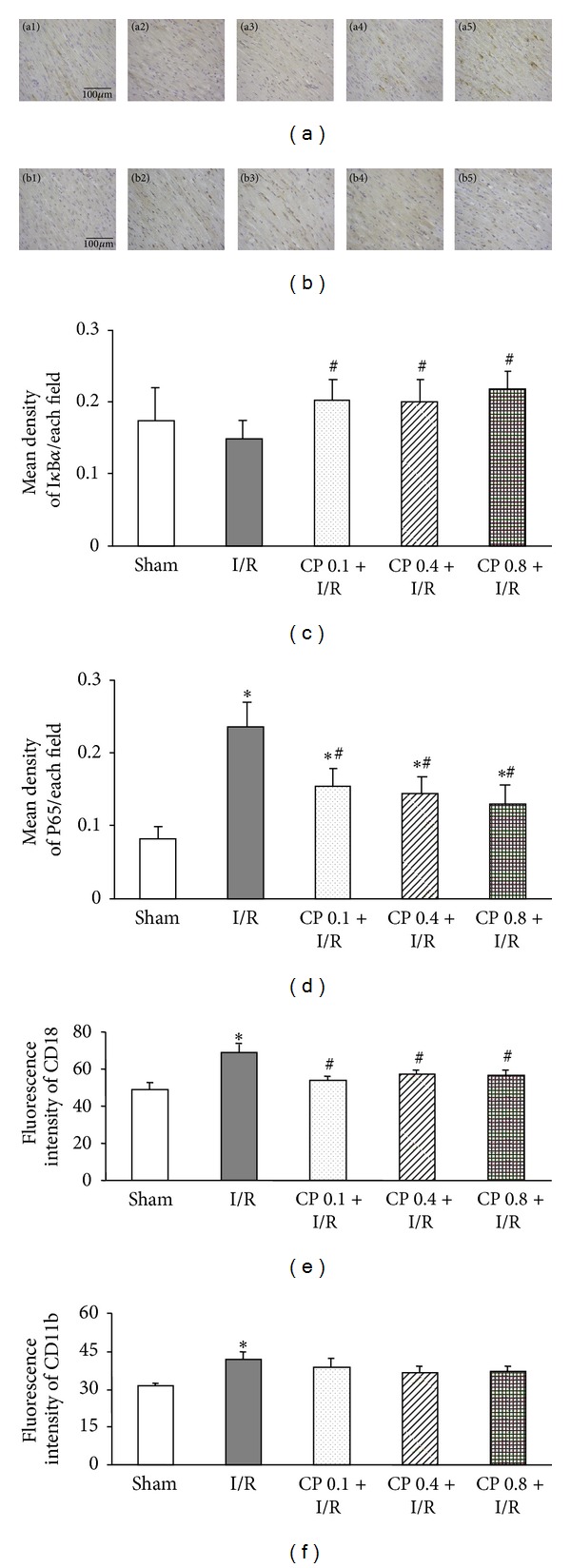
Effects of CP pretreatment on NF-*κ*B activation and adhesion molecule expression after I/R. (a) Representative images of immunohistochemistry staining for I*κ*B*α*. I*κ*B*α* positive cells are revealed by DAB (brown). Bar = 100 *μ*m. (b) Representative photographs of myocardial sections stained for P65 in different groups. P65 proteins are shown in brown color. Bar = 100 *μ*m. (c) Statistical results of I*κ*B*α* expression in five groups. (d) Quantitative analysis of P65 expression in various groups. 1, 2, 3, 4, and 5 denote Sham, I/R, CP 0.1 + I/R, CP 0.4 + I/R, and CP 0.8 + I/R group, respectively. (e) and (f) The expression of adhesion molecules CD18 and CD11b on rat neutrophils, respectively. The expression of adhesion molecules is presented as fluorescence intensity of FITC. Results are presented as mean ± SEM (*n* = 6). *P < 0.05 versus Sham group, ^#^ *P* < 0.05 versus I/R group.

 As an oxidant-sensitive transcription factor, NF-*κ*B activation plays a central role in the regulation of inflammatory response, via the augmented expression of cytokines and adhesion molecules. Activation of NF-*κ*B was assessed by detecting the expression of nuclear factor *κ*B inhibitor *α* (I*κ*B*α*) and P65 subunit of NF-*κ*B. Figures 8(a) and 8(b) display, respectively, the representative immunohistochemistry images of I*κ*B*α* and P65 around the infarct areas of left ventricular myocardium. As shown in Figure 8(c), I/R provoked a significant I*κ*B*α* degradation after 60 min of reperfusion as compared to Sham group, and administration of CP 6 days before I/R obviously prevented I*κ*B*α* degradation evoked by I/R. Supporting this result, the expression of P65 was pronouncedly upregulated by I/R compared to that in Sham group, and elevation in P65 expression was remarkably inhibited by pretreatment of CP (Figure 8(d)). 

The interaction between vascular endothelium and neutrophils is a central feature of the inflammatory response [25]. Integrins CD11 and CD18 on neutrophils are engaged in mediating neutrophil firm adhesion to endothelium and transmigration [26]. The expression of CD18 on neutrophils in blood stream was detected by flow cytometry, and the result is shown in Figure 8(e). The amount of CD18 was enhanced after I/R, which was significantly inhibited by pretreatment with CP. However, no evident difference was detected in the expression of another adhesion molecule CD11b among the five groups at 60 min of reperfusion (Figure 8(f)).

### 3.9. CP Administration Enhances Expression of Antioxidant Enzymes after I/R

**Figure 9:**
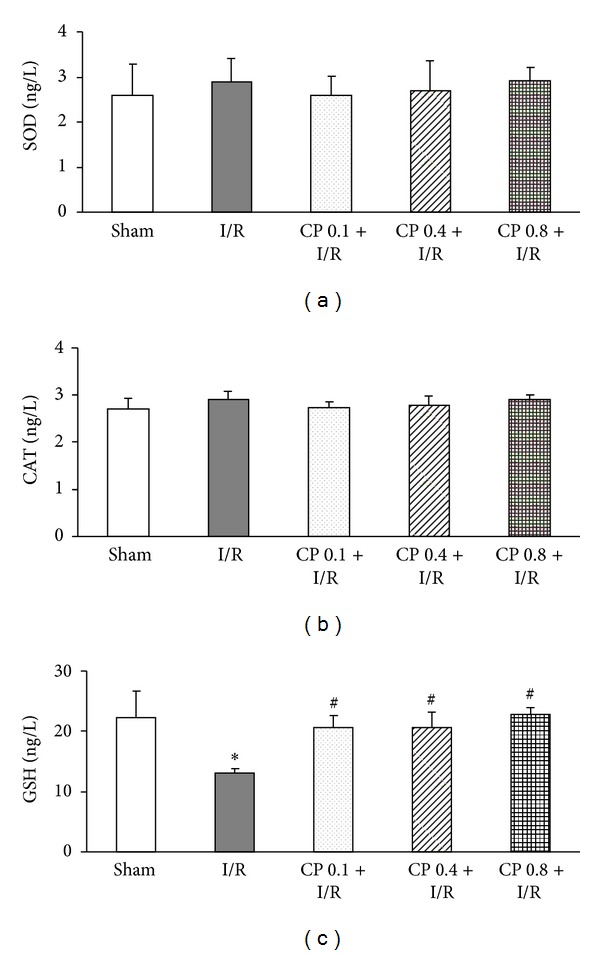
The effect of CP pretreatment on the expression of antioxidative enzymes after I/R. (a) Myocardial SOD expression in different groups. (b) The level of myocardial CAT in various groups. (c) GSH expression of myocardial tissues in five groups. Data are presented as mean ± SEM (*n* = 6). *P < 0.05 versus Sham group, ^#^ *P* < 0.05 versus I/R group.

 Endogenous antioxidant enzymes, such as SOD, CAT, glutathione peroxidase (GSH-PX), and its substrate GSH, work together to reduce free radical to water and minimize ROS-induced injury. Thus, the levels of antioxidants from myocardial tissue were tested as shown in Figure 9. There was no significant difference among the five groups at 60 min of reperfusion in the expression of SOD (Figure 9(a)) and CAT (Figure 9(b)). However, the level of myocardium GSH, a potent reductant, was dramatically reduced by exposure to I/R, which was significantly restored by pretreatment with CP at the three doses tested (Figure 9(c)).

### 3.10. CP Administration Prevents NADPH Oxidase Activation after I/R

 In the resting condition, gp91^phox^ together with p22^phox^ are located in intracellular vesicle membrane [11]. Activation of NADPH oxidase requires interactions with cytosolic organizer and modulator subunits, including p67^phox^, p47^phox^, and p40^phox^ to form an enzyme complex. Once activated, vesicles bearing NOX2 enzyme complex translocate towards and fuse with the plasma membrane [11] and transfer single electron from cytoplasmic NADPH across the plasma membrane to extracellular molecular oxygen to generate superoxide.

**Figure 10:**
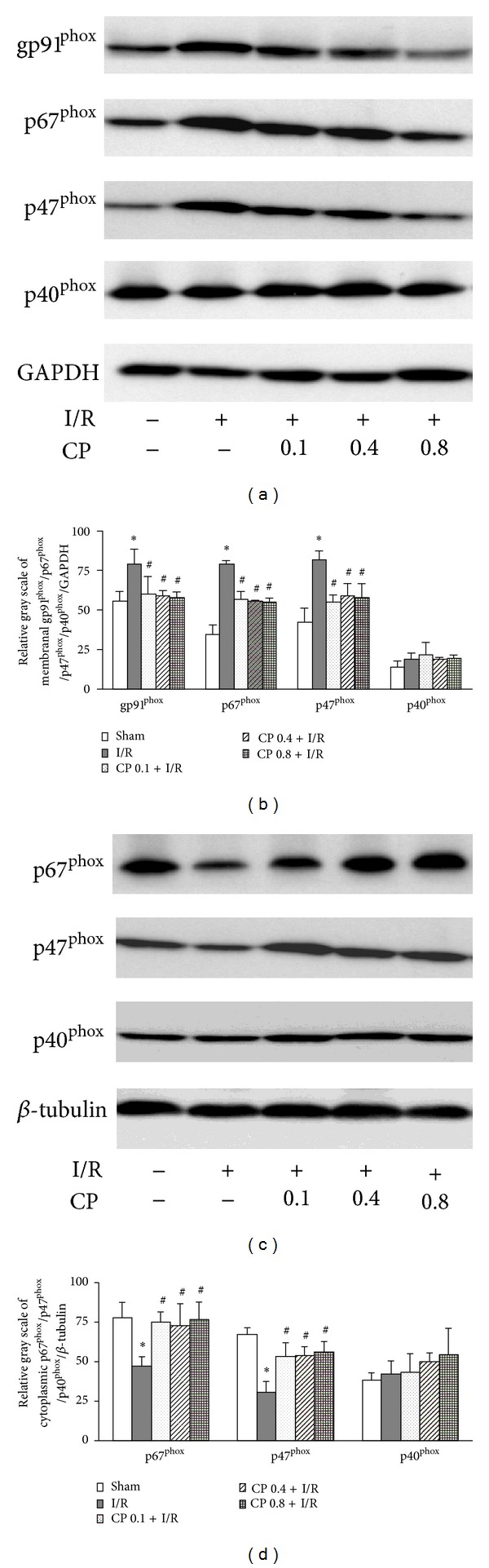
CP pretreatment inhibits NADPH oxidase activation induced by I/R. (a) Representative Western blot bands of gp91^phox^, p67^phox^, p47^phox^ and p40^phox^ on cell membrane. (b) Quantification of cell membrane expression of gp91^phox^, p67^phox^, p47^phox^ and p40^phox^. All membrane protein intensities were normalized to GAPDH. (c) Representative Western blot bands of NADPH oxidase organizer subunits p67^phox^ and p47^phox^ and modulator p40^phox^ in cytoplasm. (d) Statistical results of cytoplasm expression of p67^phox^, p47^phox^ and p40^phox^. All band intensities were calculated based on the results from 3 independent experiments. All cytosolic protein intensities were normalized to *β*-tubulin. Results are presented as mean ± SEM (*n* = 3). *P < 0.05 versus Sham group, ^#^ *P* < 0.05 versus I/R group.

To examine the activation of NADPH oxidase, expression of gp91^phox^ on the membrane and translocations of organizer subunits p67^phox^, p47^phox^, and p40^phox^ from cytoplasm to cell membrane were detected. The representative western blot bands and statistical results are shown in Figure 10. Expression of gp91^phox^, p67^phox^, and p47^phox^ on cell membrane was significantly increased after challenge by I/R compared to Sham operation, while this increase was significantly inhibited by pretreatment of CP at all three doses (Figures 10(a) and 10(b)). Correspondently, the expression of p67^phox^ and p47^phox^ in cytoplasm decreased remarkably after I/R, which was significantly blunted by pretreatment with CP (Figures 10(c) and 10(d)). On the other hand, no evident alteration in p40^phox^ expression was observed either on cell membrane or in cytoplasm, implying that p40^phox^, as a modulator, was not involved in the I/R-induced activation of NADPH oxidase. 

## 4. Discussion

In the present study, we confirm that pretreatment with CP for six days at minimal effective dose 0.1 g/kg/day significantly prevented I/R-induced myocardial infarct and apoptosis, cardiac microcirculatory dysfunction, morphological alterations, and oxidative stress. In addition, CP pretreatment dramatically inhibited I/R-induced NADPH oxidase subunits gp91^phox^ expression and p67^phox^ and p47^phox^ translocation from cytoplasm to cell membrane. Thus, this study demonstrates, for the first time, that the antioxidative activity of CP is, at least in part, dependent on its ability to inhibit NADPH oxidase activation.

Although intensive investigations have been carried out in the last decades on protecting cardiomyocytes from I/R injury, no effective therapeutic approach has been applied in clinic. One possible reason is the lack of therapeutic interventions that could simultaneously target multiple pathological events during I/R. CP is proved to be a safe and effective TCM and consisted of diverse components which are reported to relieve a variety of deleterious consequences induced by I/R, including ROS, inflammation, and apoptosis. In 2013, CP has been scheduled to undergo phase III clinical trials for prevention and treatment of ischemic cardiovascular diseases by the US Food and Drug Administration and has thus attracted increasing interest of researchers to explore the protective mechanism of CP and reveal its new clinical indications. 

CP is currently used in clinic at the dose of 0.081 g/kg/day in a multiple dosing manner. In the present study, we mimicked the multiple administration drug delivery method in clinic by giving CP for 6 days through gavage. Results show that CP was effective at 0.1, 0.4, and 0.8 g/kg/day, which were 1.2-, 4.9-, and 9.9-fold, respectively, the equivalent effective dose used in human. This result appears inconsistent with our previous results showing that a single administration of CP 90 min before I/R only at 0.8 g/kg/day, but not lower doses, attenuates cardiac microcirculation disturbance and myocardium injury after 60 min of reperfusion [17]. This discrepancy results from the difference in CP administration protocol, with a multiple dosing in the present study while a single dosing in our previous work, suggesting that CP can be applied in a manner of either a multiple dosing at low dosage, as commonly used in clinic, or of a single dosing, if necessary, at a high dosage. 

Intense inflammatory reaction during I/R has been implicated to extend myocardial injury [27]. One of the earliest inflammatory responses involves neutrophil adhesion to capillary endothelium mediated by integrins CD 18, CD 11a, and CD11b [25]. The trapped neutrophils plug the capillary and release autacoids to induce vasoconstriction and platelet aggregation [28]. In the present study, I/R caused an increase in CD18 expression on neutrophils, reduced coronary RBC velocity and myocardial blood flow, which were significantly protected by CP pretreatment, confirming the potential of CP as an anti-inflammatory agent.

Inflammatory responses are associated with increased expression of a range of cytokines and adhesion molecules, majority of which is regulated by NF-*κ*B. NF-*κ*B could be activated by cytokines themselves and free radicals as well [29]. In our experiment, we found that CP pretreatment represses the degradation of I*κ*B*α*, and inhibits the activation of NF-*κ*B. The available results suggest that this anti-inflammatory effect of CP is, at least in part, attributable to its antioxidant potential. 

I/R leads to markedly accelerated ROS production, resulting in deleterious alterations in cell structure and function. Increasing investigation has been carried out to reduce massive ROS in I/R injury. In the present study, CP pretreatment suppressed I/R-induced formation of ROS in both myocardial tissue and peripheral blood. The antioxidant activity of CP mostly, if not all, attributes to its ingredients extracted from SM. Several studies support the cardiac protective effects and oxidant scavenging activity of SM [30]. The water-soluble compounds of SM extract are mainly polyphenols, which possess aromatic ring(s) bearing one or more hydroxyl moieties [31]. Polyphenols are reported to be effective ROS scavengers due to the presence of multiple phenolic hydroxyl groups [32], which accept electron from ROS to form stable phenoxyl radicals and, in turn, interrupt the cascade of oxidation reactions in cell [33].

Oxidative stress after I/R is the result of imbalance between the massive ROS and limited antioxidant defenses. Endogenous enzymatic and nonenzymatic antioxidants include SOD, CAT, GSH-PX, and its cofactor GSH [34]. The results on the effects of CP on myocardial antioxidant expression indicated that the expression of SOD and catalase were not altered by CP administration, while an increased level of GSH was observed after 90 min of reperfusion in CP pretreated group compared with I/R group. This result suggests that, besides direct reaction with ROS by polyphenols, enhanced GSH may be another reason for the reduced oxidative stress by CP pretreatment. The ingredient(s) in CP responsible for this endpoint is unknown at present, and the mechanism details behind this effect remain to be clarified. 

For many years, researchers have focused on ROS detoxification, either using supplemented antioxidants in a nontargeted and nonspecific way like Vitamin E [35] or drugs that increase endogenous antioxidants such as GSH and SOD mimetics [36, 37]. Although much of the published results support that antioxidants protect cardiomyocytes against the damage induced by I/R, the results of several large randomized clinical trials regarding ROS scavengers in cardiovascular disease have been disappointing [38]. Thus, seeking for an approach able to directly block the enzymes involved in ROS synthesis become an attractive alternative to inhibit ROS production.

 NADPH oxidase is a main source of superoxide (O_2_^−^^·^) in most of cardiovascular diseases, which is central to the regulation of other ROS formation [39]. Massive amount of O_2_^−^^·^ produced by NADPH oxidase activates other enzymes such as xanthine oxidase (XO) and endothelial NO synthase (eNOS) to produce free radicals [40, 41]. Several cell types within heart contribute to NADPH oxidase-derived ROS production, among which vascular endothelial cells and inflammatory cells are well appreciated to produce ROS by activation of NADPH oxidase in response to I/R challenge. Regarding the role of NADPH oxidase in cardiomyocytes in I/R injury, the data so far available are controversial. Evidence in vitro showed that gene transfer of a dominant negative rac1 gene product (N17rac1) inhibits excessive ROS production in ventricular myocytes, endothelial and vascular muscle cells subjected to reoxygenation injury [42], while rac1 activation has been recognized to be the first episode of NADPH oxidase activation [39]. Moreover, elevated NOX2 expression was observed in human cardiomyocytes after acute myocardial infarction [13]. On the other hand, both NOX2^−/−^ and p47^phox−/−^ mice were not found to exhibit attenuated I/R-induced injury [43, 44]. In the current study, an enhanced activation of NADPH oxidase was revealed in cardiac tissue after I/R. We at present cannot discriminate the cell type that exhibited the activated NADPH oxidase or the relative contribution of each cell type to this outcome. Nonetheless, CP pretreatment attenuated the activation of NADPH oxidase in cardiac tissue, indicating that NADPH oxidase is one of the targets that CP acts to protect heart from I/R damage. 

Investigations have been conducted with attempt to find specific agents to inhibit the NADPH oxidase activity. For instance, peptides such as Gp91ds-tat and PR39 were shown to possess decoy p47^phox^ binding sites, which prevent interaction between p47^phox^ and NOX proteins, in turn, suppress NADPH oxidase activation [45, 46]. However, peptide drugs are limited to parenteral administration. Chemical inhibitors such as apocynin and aminoethyl benzenesulfonyl-fluoride (AEBSF) were reported to block NADPH oxidase assembly and result in a marked impairment of NADPH activation [47, 48]. However, apocynin and AEBSF have not been thoroughly defined in humans as to their specificity, efficiency, and toxicity. In the present study, we presented evidences showing the inhibitory effect of CP on NADPH oxidase activation by reducing gp91^phox^ expression and repressing translocations of p67^phox^ and p47^phox^ to cell membrane, indicating CP as a potential regime to abate the activation of NADPH oxidase during I/R.

The NADPH oxidase inhibition effect has been previously reported for the components of CP. To this end, salvianolic acid A and salvianolic acid B were shown to attenuate ROS formation and reduce the expression of gp91^phox^ and p47^phox^ in membrane fraction in hepatic fibrosis model [49]. Tanshinone II A was reported to negatively regulate upstream pathway of NADPH oxidase [50]. Similar inhibitory effects were also observed for other SM components such as rosmarinic acid [51], caffeic acid, and protocatechuic acid [52]. Ginsenoside Rb1, a major component of PN, was found to suppress oxidative damage via inhibition of NADPH oxidase subunit p47^phox^ in renal interstitial fibrosis rats [53]. The finding of the present study is in agreement with the reports above. More importantly, the results of the present study argue the superiority of CP as antioxidant over others in that it acts on multiple targets, including inhibition of NADPH oxidase, elevation of GSH, and reaction with ROS via phenolic hydroxyl groups, which collectively and efficiently reduce the oxidative stress injury to cardiac tissue after I/R. This result provides an example showing that a compound medicine may be some time preferable to a single one to cope with an insult. 

## 5. Conclusions

In summary, we reported that treatment with CP at doses ranging from 0.1 to 0.8 g/kg/day for 6 days effectively prevents rat heart from I/R-induced impacts, including infarct, apoptosis, microcirculation disturbance, oxidative stress, and inflammatory responses. Besides, CP pretreatment significantly repressed I/R-induced NADPH oxidase subunits gp91^phox^ expression and p67^phox^ and p47^phox^ translocation from cytoplasm to cell membrane. The cardioprotective effects of CP, at least partly, attribute to its antioxidative activity mediated by inhibition of NADPH oxidase activation.
